# Enhanced Electrochemical Sensing of Treprostinil: A Novel Approach for Sensitive and Selective Detection

**DOI:** 10.1021/acsomega.5c05604

**Published:** 2025-10-30

**Authors:** Çiğdem Aybüke Özata, Nevin Erk, Wiem Bouali, Asena Ayse Genc, Furkan Uzcan, Mustafa Soylak

**Affiliations:** † 37504 Ankara University, Faculty of Pharmacy, Department of Analytical Chemistry, 06560 Ankara, Turkey; ‡ Ankara University, The Graduate School of the Health Sciences, 06110 Ankara, Turkey; § 52958 Erciyes University, Faculty of Sciences, Department of Chemistry, 38039 Kayseri, Turkey; ∥ Technology Research & Application Center (TAUM), Erciyes University, 38039 Kayseri, Turkey; ⊥ Turkish Academy of Sciences (TUBA), Cankaya, 06670 Ankara, Turkey

## Abstract

Treprostinil (TRP) is a potent vasodilator used in the treatment of pulmonary arterial hypertension (PAH). The development of a rapid, sensitive, and selective electrochemical detection method for TRP is crucial for therapeutic monitoring and quality control. In this study, we present a novel electrochemical sensor for TRP based on NiPB@Cu/Cu_2_O/GCE. The NiPB@Cu/Cu_2_O/GCE sensor exhibits excellent sensitivity and selectivity, enabling reliable quantification of TRP in biological and pharmaceutical samples. The nanomaterial used in the sensor was characterized using field emission scanning electron microscopy (FE-SEM), scanning transmission electron microscopy (STEM), X-ray diffraction (XRD), and Fourier transform infrared spectroscopy (FTIR) to confirm its structural and morphological properties. The developed sensor demonstrated a wide linear range, low detection limit, and high reproducibility, making it a promising tool for clinical and pharmaceutical applications. This study provides a significant advancement in the electrochemical analysis of TRP, paving the way for further applications in drug monitoring and biomedical research.

## Graphical abstract

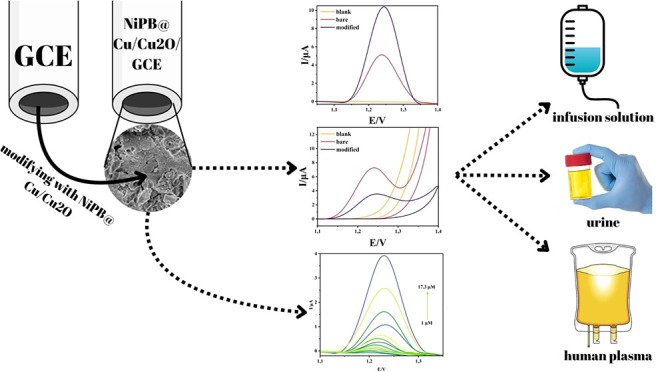

## 1 Introduction

Pulmonary arterial hypertension (PAH) is an uncommon yet severe subtype of PH, with an estimated annual occurrence of 2.4 cases per million people in the United States^1^ and the estimated incidence in Europe is comparable.^2^ It involves elevated blood pressure in the arteries carrying blood from the heart to the lungs, with the resulting increase in pulmonary vascular resistance potentially leading to right ventricular failure and making the condition both progressive and life-threatening.^1^ Pulmonary arterial hypertension (PAH) is still incurable, but since the first targeted therapy was approved in 1995, many new medications have been introduced, most of which work as pulmonary vasodilators.^3^ Synthetic prostacyclin (PGI2) and its analogs are among the most effective treatments for PAH patients, but their broader use is hindered by challenges in administration and other constraints.^1^

Treprostinil (TRP) is a tricyclic prostacyclin analog known for its chemical stability,^4^ with a molecular formula of C_23_H_34_NaO_5_ and a molecular weight of 390.52.^5^ Treprostinil reduces pulmonary artery pressure mainly by inducing direct vasodilation in both pulmonary and systemic arterial vascular networks. This enhances systemic oxygen delivery and boosts cardiac output while causing minimal changes to heart rate.^6^

TRP is available in intravenous (IV), subcutaneous, oral, and inhaled forms, offering flexibility in dose adjustment and administration routes.^7^ The Food and Drug Administration (FDA) approved treprostinil in 2009 for inhaled use, in 2013 for oral administration, and in 2017 for infusion therapy.^8^ While treprostinil (TRP) has shown clinical benefits, its rapid elimination requires continuous infusion or frequent dosing, which can diminish its therapeutic advantages due to the occurrence of adverse events with prolonged or repeated administration.^9^ TRP’s adverse effects vary based on the route of administration but commonly include headache, flushing, nausea, diarrhea, jaw pain, and muscle or joint pain. Subcutaneous administration is often associated with severe pain and redness at the injection site,^10^ while intravenous use carries a higher risk of infections, such as bloodstream infections, due to the need for central venous catheters.^11^ Inhaled treprostinil can cause cough, throat irritation, dizziness, and shortness of breath,^12^ whereas the oral form may lead to gastrointestinal discomfort, including abdominal pain, nausea, and vomiting.^13^ Treprostinil dosing is individualized to optimize therapeutic efficacy while minimizing adverse effects. Initiation typically involves a low starting dose, followed by gradual titration under close medical supervision to reduce the risk of side effects. Regular clinical monitoring is essential to evaluate treatment effectiveness and identify potential complications.

The determination of treprostinil after it is administered to the human body is crucial to ensuring both the effectiveness of the treatment and the safety of the patient. Close observation during dose titration helps minimize side effects, improving the tolerability of the medication and supporting the success of the therapy. This is particularly important for intravenous or subcutaneous infusion methods, where the risks of infections and site-specific reactions need to be carefully managed. Regular follow-up, tailored to the patient’s lifestyle and compatibility with the treatment method, ensures sustained therapeutic benefits while preventing potential complications. Given these considerations, reliable analytical techniques are essential for monitoring the administered dose of treprostinil (TRP) in patients.

A comprehensive review of the literature revealed that, to date, only a couple of studies have been conducted on the analytical determination of treprostinil, utilizing the liquid chromatography-tandem mass spectrometry method (LC-MS/MS)^14^ and reverse phase-high performance liquid chromatography (RP-HPLC).^15,16^ Chromatography is a highly sensitive technique, but it has some drawbacks, including high equipment and maintenance costs, the need for specialized training, and time-consuming sample preparation.^17^ In contrast, electrochemical techniques have been designed for the analysis of numerous significant analytes, providing benefits such as user-friendliness, rapid response times, reduced instrumentation costs, enhanced sensitivity, and accurate analytical performance.^18^

The field of electroanalysis continues to evolve, with a growing variety of electrodes being developed and existing ones undergoing continuous refinement. Glassy carbon electrodes (GCEs) are particularly notable among carbon-based electrodes because of their outstanding capacity for surface functionalization, effortless surface renewal, and high reproducibility.^19^ GCEs offer several advantages that make them highly suitable for electrochemical applications. Their excellent chemical stability guarantees stable performance across various chemical media, while their large surface area improves the responsiveness of electrochemical processes. Furthermore, the minimal background current of these sensors contributes to improving the sensitivity thresholds, allowing for the determination of even low concentration of the target analyte. Additionally, their superior electrical conductivity enables rapid signal transmission, thereby enabling fast and precise analytical measurements.^20^ Moreover, GCEs demonstrate excellent biocompatibility, making them highly suitable for utilize in processes involving complex matrices. Their remarkable repeatability, combined with the simplicity of surface customization, further enhances their cost-effectiveness and versatility, allowing them to be effectively applied in a wide range of sensor technologies and related fields.^21^

Prussian Blue (PB) is a widely utilized compound in various applications, not only in sensors but also in electrochromic devices and electrocatalytic electrodes.^22–24^ Chemically, PB is known as ferric hexacyanoferrate (Fe_4_[Fe­(CN)_6_]_3_). It is a prominent anodic electrochromic material capable of transitioning from blue to transparent, exhibiting high optical contrast, excellent chemical stability, and a rapid response time.^25^

Prussian Blue analogues (PBAs), generally represented as A_*x*_M_*y*_[M′(CN)_6_]­z, where A denotes an alkali metal cation and M and M′ are transition metals, have attracted significant research interest due to their versatile electrochemical properties.^26^ Incorporating nickel (Ni) into PBAs, particularly in nickel hexacyanoferrates, allows for fine-tuning of their voltage and capacity by adjusting the composition. This strategy has the potential to enhance the electrochemical performance of PBAs by mitigating limitations such as undesirable phase transitions during cycling, thereby improving structural stability and long-term durability.^27^

It is widely recognized that copper nanomaterials have garnered significant attention due to their abundance, wide availability, and low cost compared to other nanomaterials.^28^ Copper-based nanomaterials, in both their metallic and oxidized forms, have attracted significant attention in electrochemical sensor applications and various other fields. The combination of metallic copper (Cu) and its oxide form (Cu_2_O) creates a synergistic effect, enhancing their electrochemical performance in different applications, owing to their redox activity, electrical conductivity, and surface characteristics.^29–31^

Despite their individual advantages, the direct combination of Ni-based PBAs with Cu/Cu_2_O has been scarcely investigated in the context of electrochemical sensing. Previous studies have focused either on the electrocatalytic behavior of nickel-based Prussian Blue analogs or on copper/cuprous oxide systems separately; however, their integrated structure remains largely unexplored, especially for pharmaceutical analysis.^32–34^

The rationale behind merging NiPB and Cu/Cu_2_O lies in exploiting the complementary properties of both materials: NiPB contributes high ion-exchange capacity and stable redox transitions, while Cu/Cu_2_O offers superior conductivity and active surface area. This commodification is expected to provide a heterostructure with faster charge transfer kinetics, improved electrocatalytic activity, and enhanced analytical performance—a synergy that has been theoretically discussed but rarely validated in practical sensor development.^35^

To the best of our knowledge, no prior study has reported the application of a NiPB@Cu/Cu_2_O-modified electrode for the detection of treprostinil or any prostacyclin analog. This combination introduces a novel sensing architecture that bridges redox-based ion-exchange materials with conductive nanostructures for sensitive drug monitoring.

This research aims to design an innovative electrochemical sensor for the sensitive and selective determination of treprostinil (TRP). By modifying a GCE with a NiPB@Cu/Cu_2_O composite, the sensor’s electrochemical performance was systematically evaluated using DPV and CV. The study seeks to optimize sensor conditions, assess its electrocatalytic activity, and investigate its analytical capabilities, including selectivity, repeatability, and spiked-sample analysis in plasma, urine, and pharmaceutical formulations. This research represents the first electrochemical approach for TRP detection, aiming to offer a cost-effective, rapid, and reliable alternative to existing analytical methods.

## 2 Experimental Section

### 2.1 Experimental Materials and Instruments

The Supporting Material provides details on the experimental materials and instruments.

### 2.2 Preliminary Treatment and Surface Preparation of the Electrode

The preparation of the NiPB@Cu/Cu_2_O/GCE began with polishing the electrode on a polishing pad using a 0.05 μm alumina slurry. Afterward, the electrode was thoroughly rinsed with ethanol, followed by a distilled water wash, and subsequently air-dried. The pretreated GCE was then utilized for the modification process with the synthesized composite suspension. Specifically, 2.0 mg of the prepared composite was dispersed in 2 mL of distilled water and subjected to sonication for approximately 15 min to ensure uniform dispersion. An optimized volume of 4.0 μL of this composite suspension was carefully drop-cast onto the surface of the GCE. The modified electrode was then dried using an infrared lamp (8 min), rendering it ready for use as a modified GCE in electrochemical studies.

### 2.3 Spiked Sample Pretreatment for TRP

#### 2.3.1 Plasma

In order to precipitate proteins in the human plasma, 1 mL of acetonitrile was added to 1.0 mL of a fresh plasma sample. The mixture was centrifuged at 8000 rpm for 20 min. After protein precipitation, a suitable amount of the supernatant layer was carefully moved into a volumetric flask to obtain the protein-free human serum and adjusted to the target concentration with TRP solution to reach the required volume. Recovery studies were conducted by utilizing calibration curve data and spiking specific concentrations of pure TRP solution. For each concentration, the relative standard deviation (RSD) values were determined.

#### 2.3.2 Urine

Urine specimens were obtained from healthy volunteers, and the spiked urine sample was filtered through a PTFE membrane filter (0.45 μm). After that, the filtered urine was combined with different concentrations of TRP solutions. The recovery studies were carried out using the data in calibration curves and adding pure TRP solution to certain concentrations, and relative standard deviation (RSD) values were calculated for each concentration.

#### 2.3.3 Pharmaceutical Sample

Trepoks infusion solution, separated from its excipients, was diluted with 0.1 M B-R solution at the desired ratio. Then, it was mixed with different concentrations of the TRP solution and analyzed by the standard addition method.

### 2.4 Synthesis of NiPB@Cu/Cu_2_O

Na_2_Ni­[Fe­(CN)_6_] (nickel-based Prussian blue analog, NiPB) was synthesized using previously reported methods.^36^ In the first step, Ni_2_·4H_2_O^37^ (2.69 g, 10 mmol) was dissolved in a solvent mixture consisting of 175 mL deionized water and 25 mL DMF to prepare Solution A. Concurrently, Solution B was prepared by dissolving Na_4_[Fe­(CN)_6_]·10H_2_O (4.84 g, 10 mmol) and NaCl (7 g, 0.12 mol) in 175 mL deionized water. Solution A was then introduced dropwise into Solution B under constant mechanical stirring at room temperature, and the reaction was allowed to proceed for 72 h. The resulting colloidal suspension was centrifuged to isolate the solid product, which was subsequently washed three times with ethanol to remove impurities. The purified product was then dried at 50 °C for 12 h in a vacuum oven to obtain the final NiPB material. This synthesis route ensures the formation of a well-defined nickel-based Prussian blue analogue with high purity and consistency.

The NiPB@Cu/Cu_2_O composite was fabricated through a sodium borohydride reduction process.^38^ In a a standard preparation, Cu­(CH_3_COO)_2_·H_2_O (0.8 mmol) and NiPB (0.5 g) were dispersed in deionized water (10 mL) and stirred for 30 min to create a uniform suspension. In a separate step, a reducing solution was prepared by dissolving NaBH_4_ (0.075 g) and NaOH (0.075 g) in deionized water (1 mL). This reducing solution was then introduced dropwise into the metal salt suspension at a controlled rate of 0.1 mL min^–1^ while maintaining vigorous agitation. The reaction was allowed to proceed for 1 h under continuous stirring to ensure thorough mixing and complete reduction. The resulting solid product was separated by centrifugation at 8000 rpm (3 min) employing a high-speed centrifuge. To eliminate any residual impurities, the collected solid was subjected to ten cycles of washing with alternating ethanol and deionized water. After purification, the final product was dried in a vacuum oven at 50 °C for 8 h, resulting in the formation of the NiPB@Cu/Cu_2_O composite. It is worth noting that Cu/Cu_2_O separate was also synthesized using the same method, following identical steps but without the addition of NiPB. This synthesis protocol ensures the production of high-purity materials with well-defined properties, making them suitable for various advanced applications.

## 3 Results and Discussion

### 3.1 Characterization of NiPB@Cu/Cu2O

#### 3.1.1 Fourier-Transform Infrared Spectroscopy (FTIR) Analysis

The FTIR spectrum, as shown in Figure 1A­(a), corresponds to the NiPB material and exhibits an absorption peak at approximately 2085 cm^–1^, which is assigned to the C≡N stretching vibration. Additionally, absorption bands are observed at 1440 cm^–1^ and 1339 cm^–1^, related to the symmetric stretching of carboxylate groups and −CH deformation, respectively. Furthermore, absorption bands at 592 cm^–1^ and 468 cm^–1^ are identified, which are associated with Fe–CN bending and Fe–CN stretching vibrational modes, respectively. These spectral features provide clear evidence of the chemical structure and bonding characteristics of the NiPB material.^39^

**Figure 1:**
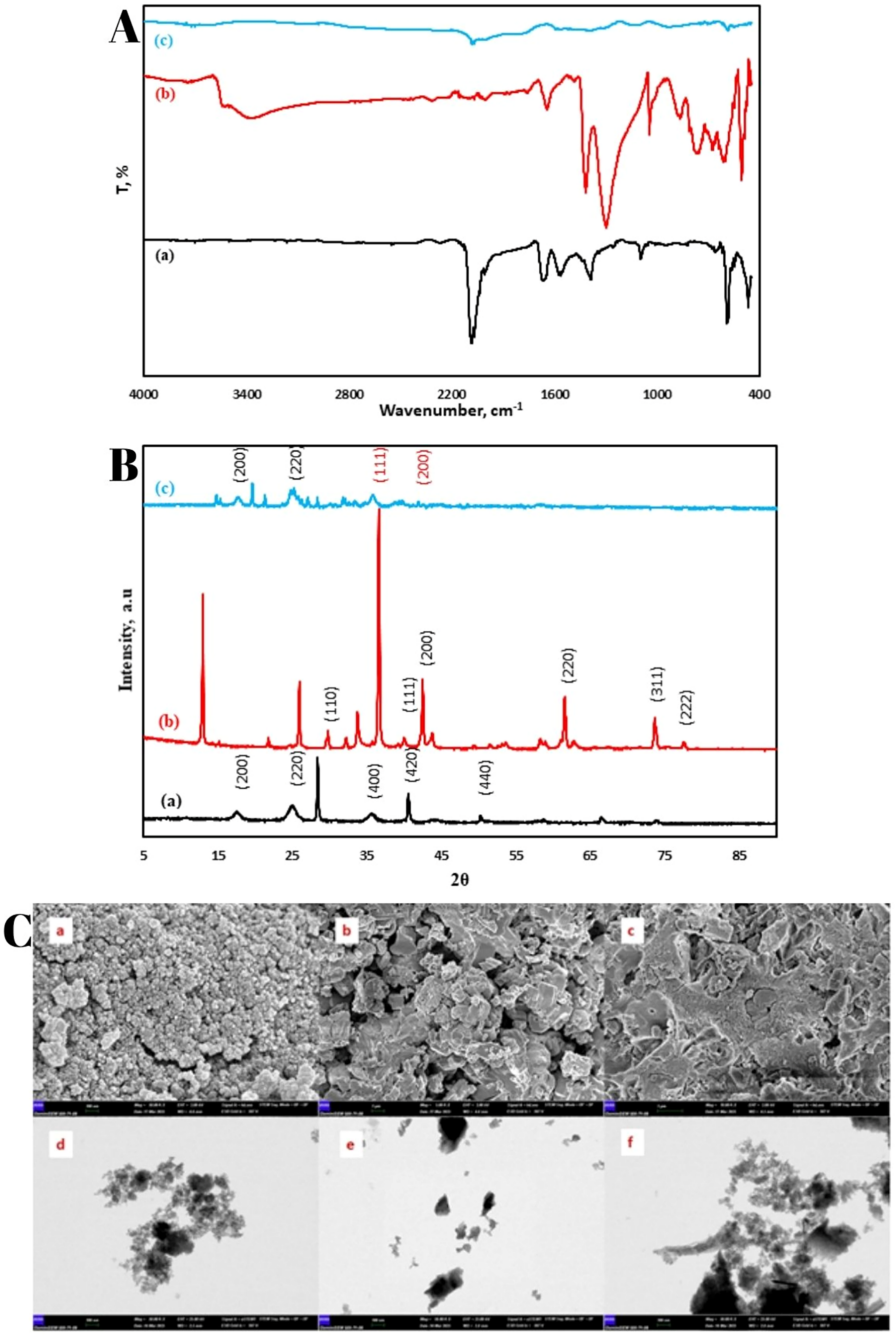
FT-IR spectrum of NiPB A­(a), Cu/Cu_2_O A­(b) and NiPB@Cu/Cu_2_O A­(c). XRD data of NiPB B­(a), Cu/Cu_2_O B­(b) and NiPB@Cu/Cu_2_O B­(c). FE-SEM images of (NiPB C­(a), Cu/Cu_2_O C­(b) and NiPB@Cu/Cu_2_O C­(c)) and STEM images of (NiPB C­(d), Cu/Cu_2_O C­(e) and NiPB@Cu/Cu_2_O C­(f)).

The FTIR results for Cu/Cu_2_O, as depicted in Figure 1A­(b), display a distinct and sharp absorption peak at 603 cm^–1^ in the spectrum of Cu/Cu_2_O. This peak is attributed to vibrational mode of Cu­(I)–O, thereby verifying the identification of crystalline Cu_2_O in the synthesized material. This characteristic peak serves as a key indicator of the structural composition and crystallinity of the Cu/Cu_2_O composite.^38^

The structure of the NiPB@Cu/Cu_2_O composite incorporates features from both components, as clearly illustrated in Figure 1A­(c). The FTIR spectrum of the composite reveals a sharp absorption peak at 603 cm^–1^, characteristic of the Cu­(I)–O lattice vibration in the Cu/Cu_2_O spectrum, verifying the presence of crystalline Cu_2_O. Additionally, the spectrum exhibits an absorption peak at approximately 2085 cm^–1^, which is ascribe to the C≡N stretching vibration of the NiPB material. These distinct spectral features demonstrate the successful integration of both NiPB and Cu/Cu_2_O within the composite structure.^38,39^

#### 3.1.2 X-ray Diffraction Analysis

The XRD results for NiPB, as depicted in Figure 1B­(a), reveal characteristic diffraction peaks at 2θ = 17°, 24°, 35°, 39°, and 50°. These peaks correspond to the (200), (220), (400), (420), and (440) crystallographic planes of NiPB, respectively, as confirmed by the JCPDS card no. 51–1987. These well-defined peaks indicate the crystalline nature and phase purity of the NiPB material, consistent with its expected structural properties.^40^

The XRD pattern of Cu/Cu_2_O, as illustrated in Figure 1B­(b), demonstrates distinct diffraction peaks at 2θ = 30°, 37°, 43°, 62°, 74°, and 78°. These peaks correspond to the crystallographic planes (110), (111), (200), (220), (311), and (222), respectively, and align well with the reference ICDD #01—078—2076. The presence of these well-defined peaks confirms the successful synthesis of the Cu/Cu_2_O nanocomposite at ambient temperature utilizing a straightforward aqueous chemical solution method. This result highlights the crystalline nature and phase purity of the synthesized material.^41^

The XRD pattern of the NiPB@Cu/Cu_2_O composite, as shown in Figure 1B­(c), reveals the successful integration of both NiPB and Cu/Cu_2_O phases. Characteristic peaks from NiPB are observed at 2θ = 17° and 24° corresponding to the (200) and (220) planes, respectively. Additionally, peaks from Cu/Cu_2_O are identified at 2θ = 35° and 43°, which correspond to the (111) and (200) planes, respectively. Notably, the peak at 35° is shared between both NiPB and Cu/Cu_2_O, indicating the coexistence of the two phases within the composite. The presence of these distinct and overlapping peaks, confirms the successful synthesis of the NiPB@Cu/Cu_2_O composite. This result demonstrates the effective combination of both materials into a single composite structure, preserving the crystalline properties of each component.^40,41^

The FE-SEM images (Figure 1C­(a–c)) and STEM images (Figure 1C­(d–f)) reveal the distinct morphologies of NiPB, Cu/Cu_2_O, and NiPB@Cu/Cu_2_O nanoparticles, respectively. The NiPB nanoparticles display a porous and irregular surface texture, indicative of a high surface area that can significantly enhance its reactivity, making it highly suitable for a wide range of applications. On the other hand, the Cu/Cu_2_O nanocomposite exhibits a granular and tightly packed morphology, reflecting a more dense and uniform structure. The NiPB@Cu/Cu_2_O nanocomposite, however, demonstrates a more consolidated and integrated morphology compared to its individual components. The incorporation of Cu/Cu_2_O appears to influence the structural properties of NiPB, leading to a more cohesive and compact composite material. These morphological variations underscore the successful combination of NiPB and Cu/Cu_2_O into a unified composite, effectively merging the beneficial characteristics of both materials.

### 3.2 Evaluation of Electrocatalytic Performance of the NiPB@Cu/Cu_2_O/GCE

The electrocatalytic properties of the NiPB@Cu/Cu_2_O/GCE were systematically evaluated by focusing on two primary factors: the electrochemically active surface area and the reduced charge-transfer resistance.

The electrochemical performance of both electrodes was assessed using CV in a 0.1 M KCl solution with 5 mM [Fe­(CN)_6_]^3–^/^4–^ at a scan rate of 50 mV/s, as illustrated in Figure 2A. The NiPB@Cu/Cu_2_O/GCE displayed a markedly higher redox peak current and a reduced peak-to-peak separation (Δ*E*_p_ = *E*_pc_ – *E*_pa_) of approximately 27 mV, indicating enhanced electrocatalytic activity. Furthermore, the electroactive surface areas (ESA) of the bare GCE and NiPB@Cu/Cu_2_O/GCE were determined by analyzing their CV responses in a solution of [Fe­(CN)_6_]^3–^/^4–^ (5 mM) and KCl while adjusting the scan rate (ν) between 0.05 and 0.3 V/s. The peak currents (*I*_pa_) showed a linear correlation with the square root of the scan rate (ν^1/2^, *R* ^2^ > 0.99) for both bare GCE and NiPB@Cu/Cu_2_O/GCE, confirming that the electron transfer mechanism at these electrodes is governed by diffusion. Additionally, the log­(*I*_p_)–log­(ν) plots yielded slopes of ∼0.3, further supporting a predominantly diffusion-controlled process (Figures S1–S2). Based on the Randles–Sevcik eq (eq S1), the slopes of the *I*_pa_ vs v^1/2^ plots the ESA were found to be 0.07 cm^2^ for the bare GCE and 0.10 cm^2^ for the NiPB@Cu/Cu_2_O/GCE. These results confirm that the NiPB@Cu/Cu_2_O/GCE possesses a larger ESA, offering an increased number of reactive sites.

**Figure 2:**
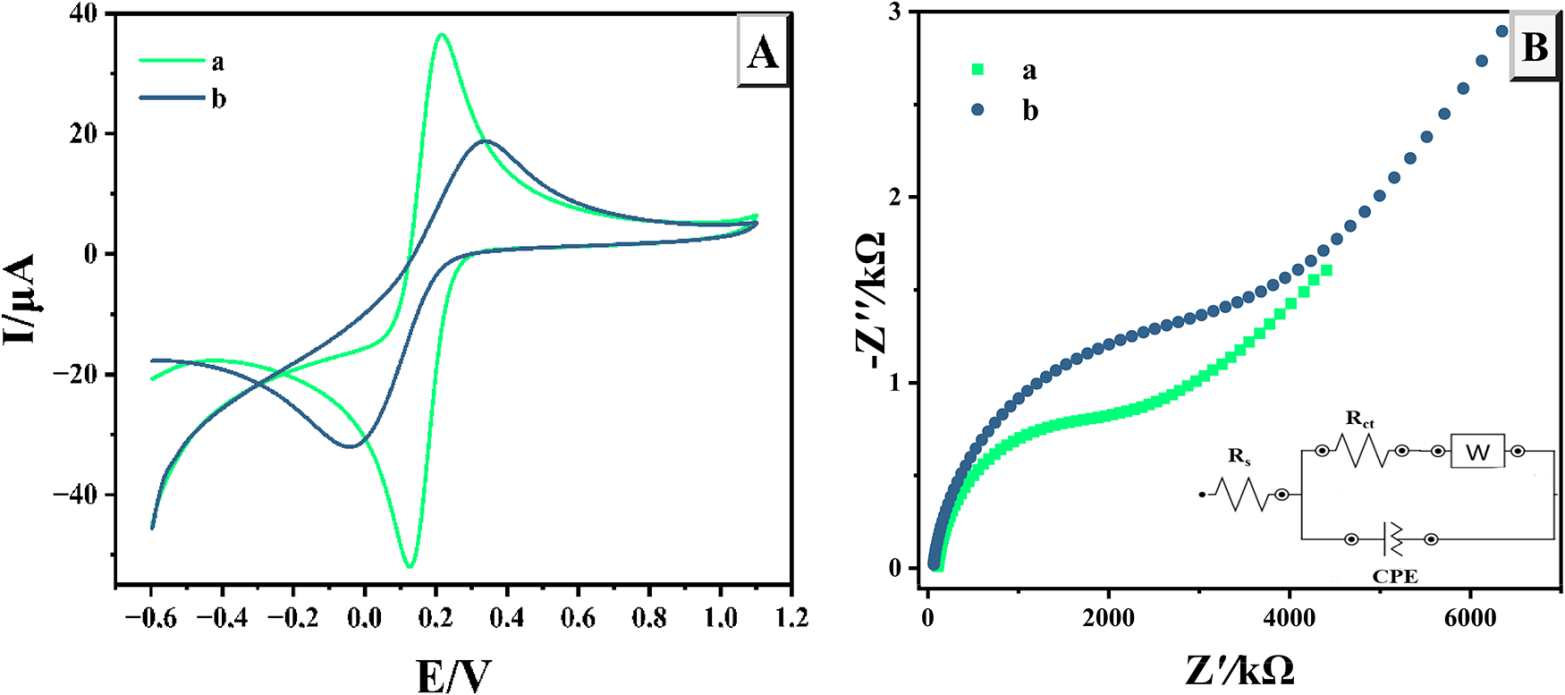
CV (A) and EIS (B) for the NiPB@Cu/Cu_2_O/GCE (a) and unmodified electrode (b) in [Fe­(CN)_6_]^3–/4–^ (5 mM) and KCl.

Electrochemical impedance spectroscopy (EIS) was employed as a powerful analytical technique to investigate the interfacial properties of both the modified and unmodified electrodes. Figure 2B presents Nyquist plots comparing the electrochemical behavior of the NiPB@Cu/Cu_2_O-modified electrode with that of the unmodified GCE in 5 mM [Fe­(CN)_6_]^3–^/^4–^. The Nyquist plots exhibit two distinct regions: a semicircular component observed at high frequencies and a linear segment at low frequencies. The semicircle in the high-frequency region corresponds to the electron-transfer process, with its diameter representing the charge-transfer resistance (*R*_ct_). A smaller semicircle diameter for the NiPB@Cu/Cu_2_O/GCE compared to the bare GCE suggests a significant reduction in charge-transfer resistance, indicating enhanced electron transfer kinetics at the electrode surface. In contrast, the low-frequency linear portion of the Nyquist plot is indicative of a diffusion-controlled process, which reflects the mass transport of redox species to the electrode interface. These findings highlight the superior electrochemical performance of the NiPB@Cu/Cu_2_O-modified electrode, demonstrating its potential for improved charge transfer efficiency and enhanced analytical sensitivity.

A significant difference in *R*_ct_ values between the bare and modified electrodes provides evidence of surface modification. The bare GCE exhibits an *R*_ct_ of approximately 4475.8 Ω, while the modified electrode demonstrates a much lower value of 2184.6 Ω (Figure 4B), indicating that the modified surface exhibits enhanced conductivity and promotes faster electron transfer.

The electron transfer rate constant (*k*_0_) and exchange current density (*j*_0_) for both electrodes were calculated employing eqs S2 and S3 (Table 1). The *k*_0_ value for the NiPB@Cu/Cu_2_O/GCE (2.36 × 10^–4^ cm s^–1^) was higher than that of the bare GCE (1.54 × 10^–4^ cm s^–1^), confirming the modified electrode’s superior ability to facilitate electron transfer and improve overall electrochemical performance. Similarly, the exchange current density (*j*_0_) for the NiPB@Cu/Cu_2_O/GCE was determined to be 11.3 × 10^–4^ A cm^–2^, significantly exceeding that of the bare electrode (7.4 × 10^–4^ A cm^–2^). This enhancement can be attributed to the increased surface area and additional functional groups present on the modified electrode. These results align well with the findings obtained from CV analysis, further demonstrating the enhanced electrochemical performance of the modified electrode. Consequently, the NiPB@Cu/Cu_2_O/GCE shows strong potential for applications in electrochemical sensing.

**Table 1:** Electrochemical Parameters Measurements on Bare and NiPB@Cu/Cu_2_O/GCE

electrode	Δ*E*_p_ (V)	ESA (cm^2^)	*k* ^0^ (cm s^–1^)	*j*_0_ (A cm^2^)
GCE	0.35	0.07	1.54 × 10^–4^	7.4 × 10^–4^
NiPB@Cu/Cu_2_O/GCE	0.08	0.10	2.36 × 10^–4^	11.3 × 10^–4^

### 3.3 Electrochemical Properties of TRP at the Surface of Prepared NiPB@Cu/Cu_2_O/GCE

Choosing a suitable electrode is crucial in the electrochemical determination of analyte molecules. In this study, The modification of the sensor was verified by comparing the CV and DPV responses of unmodified GCE, and NiPB@Cu/Cu_2_O/GCE.

Under experimental conditions in 0.1 M Britton–Robinson (B–R) buffer at pH 2.0, both the bare GCE and the NiPB@Cu/Cu_2_O/GCE showed no oxidation peaks in the absence of TRP (Figure 3A, curve a). However, in the presence of TRP, the unmodifed GCE exhibited an anodic peak at 1.23 V with a current of 5.25 μA as shown in Figure 3A, curve b. In contrast, the NiPB@Cu/Cu_2_O/GCE demonstrated enhanced electrochemical performance, showing an increased oxidation peak current (10.74 μA) and a slight shift in potential (1.25 V) as illustrated in Figure 3A, curve c, suggesting that the composite modification improved the sensor’s electrocatalytic activity toward TRP oxidation.

**Figure 3:**
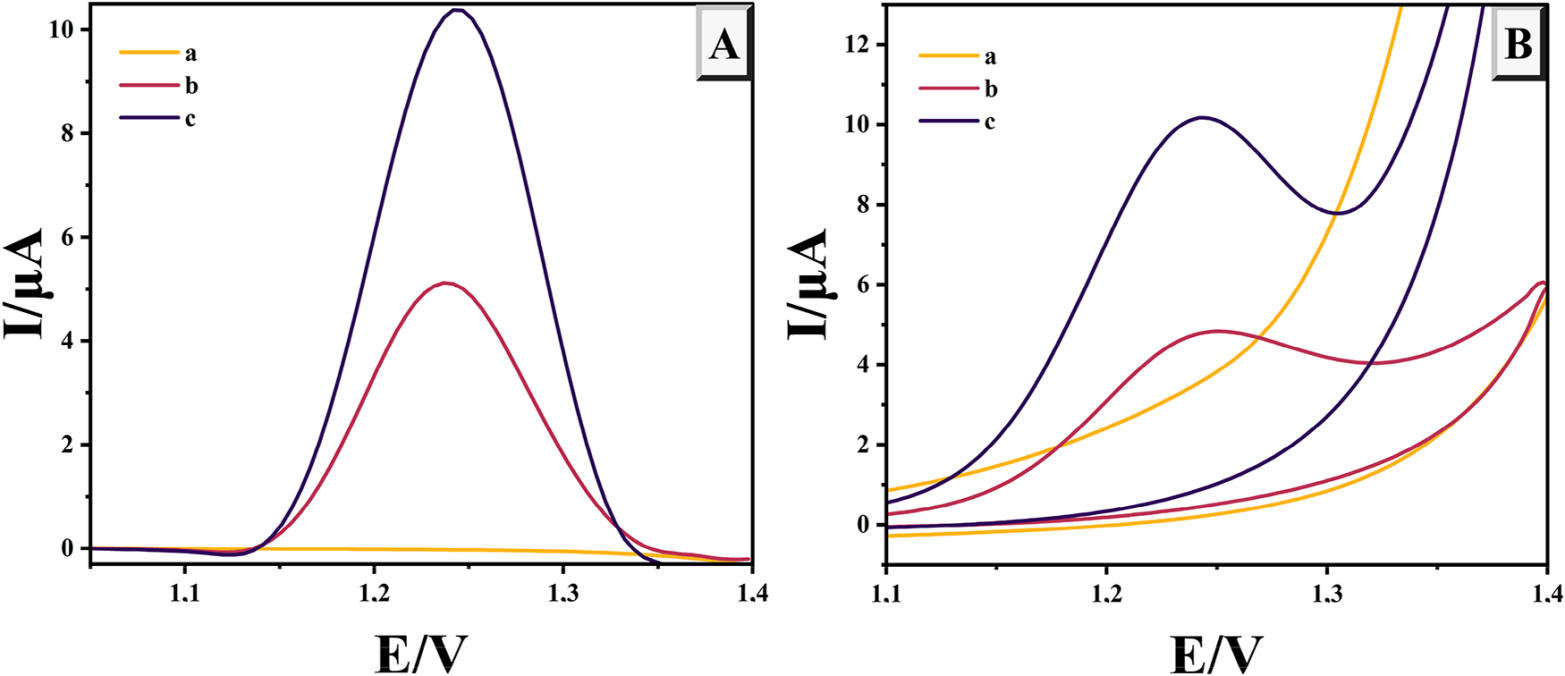
DPV (A) and CV (B) voltammograms obtained by bare GCE and NiPB@Cu/Cu_2_O/GCE in the absence and presence of 50 μM TRP in a solution of 0.1 M B–R (pH = 2.0) (a: blank, b: bare electrode, c: NiPB@Cu/Cu_2_O-modified electrode).

Further analysis using CV revealed that neither the bare GCE nor the NiPB@Cu/Cu_2_O/GCE displayed redox activity in 0.1 M B–R buffer (pH 2.0) in the absence of TRP (Figure 3B, curve a). Upon the addition of TRP, the unmodifed GCE produced an irreversible oxidation peak at 1.24 V with a current of 2.31 μA as demonstrated in Figure 3B, curve b. After modification with the composite material, the oxidation peak current significantly increased to 4.98 μA, accompanied by a slight negative potential shift to 1.23 V (Figure 3B, curve c). This increase in current, coupled with the reduction in oxidation potential, highlights the electrocatalytic properties and high conductivity of the composite material on the modified electrode.

Moreover, the DPV method demonstrated superior sensitivity for TRP detection compared to CV, as evidenced by the enhanced oxidation peak current. Therefore, DPV was chosen as the optimal technique for TRP detection in this study.

### 3.4 Evaluation of Ideal Operating Conditions

#### 3.4.1 Investigating Electrolyte and pH Effects for Improved Electrochemical Sensing

The choice of supporting electrolyte plays a critical role in determining the solution’s conductance and energy efficiency. To identify the most suitable electrolyte, several options, such as phosphate-buffered saline (PBS), Britton–Robinson (B–R) buffer, hydrochloric acid (HCl), and potassium chloride (KCl) were evaluated utilizing the DPV approach in the presence of 50 μM TRP. As depicted in Figure S3, the B–R buffer at pH 2.0 provided the highest oxidation peak current, making it the preferred electrolyte for further experiments.

The pH of the electrolyte also significantly affects electrochemical detection by altering the oxidation peak current. For optimal sensor design, material selection should consider these pH-dependent changes. Figure S4 shows the influence of solution pH on the electrocatalytic peak current of TRP at NiPB@Cu/Cu_2_O/GCE in a 0.1 M B–R solution, analyzed using DPV over a pH range of 2.0 to 5.0. The results indicated that pH 2.0 yielded the most favorable peak current for TRP, leading to its selection for subsequent studies.

#### 3.4.2 Determining the NiPB@Cu/Cu_2_O/GCE Concentration and Quantity

In order to develop an electrochemical sensor with enhanced performance for TRP analysis, the experimental conditions were carefully optimized by varying the amount and concentration of the NiPB@Cu/Cu_2_O/GCE composite.

To investigate the effect of NiPB@Cu/Cu_2_O/GCE concentration on the peak current of 50 μM TRP, solutions with concentrations ranging from 0.5 to 2.0 mg/mL were prepared and subsequently applied in appropriate amounts to modify the GCE.

Afterward, to examine the influence of the composite volume, 2 to 6 μL of the NiPB@Cu/Cu_2_O/GCE were applied to the surface of the GCE in a B–R buffer solution at pH 2.0. After conducting these experiments, the ideal conditions were identified as a composite volume of 4 μL and a concentration of 1.0 M, as shown in Figure S6A,B.

#### 3.4.3 Analyzing the Electrochemical Response of TRP at Different Scan Rates

The impact of scan rate on the electro-oxidation of 50 μM TRP was investigated using cyclic voltammetry (Figure 4A). A linear relationship was observed between the square root of the scan rate and the peak current within the range of 10 to 75 mV, indicative of a diffusion-controlled process. This relationship can be described by the equation *I*_pa_ = 0.179υ^1/2^ + 0.097 with a coefficient of determination (*R* ^2^) of 0.993 (Figure 4B). Furthermore, the correlation between log *I*_pa_ and log υ was also linear, expressed as log *I*_pa_ = 0.540 log υ – 0.855, with *R* ^2^ = 0.991 (Figure 4C). The slope of 0.540 is consistent with the theoretical value of 0.5 for diffusion-controlled processes, thereby reinforcing the conclusion that the electro-oxidation of TRP is governed by diffusion. Additionally, an increase in scan rate resulted in a positive shift of the peak potential. the peak potential exhibited a good linear relationship with the natural logarithm of the scan rate over the same range (10 to 75 mV), represented by the equation *E*_pa_ = 0.044 ln υ + 1.050 (*R* ^2^ = 0.998) (Figure 4D). By applying Laviron’s theory to irreversible electrode reactions (eq S4), the electron count involved in the oxidation process of TRP was calculated to be 1.167 (∼1.0).

**Figure 4:**
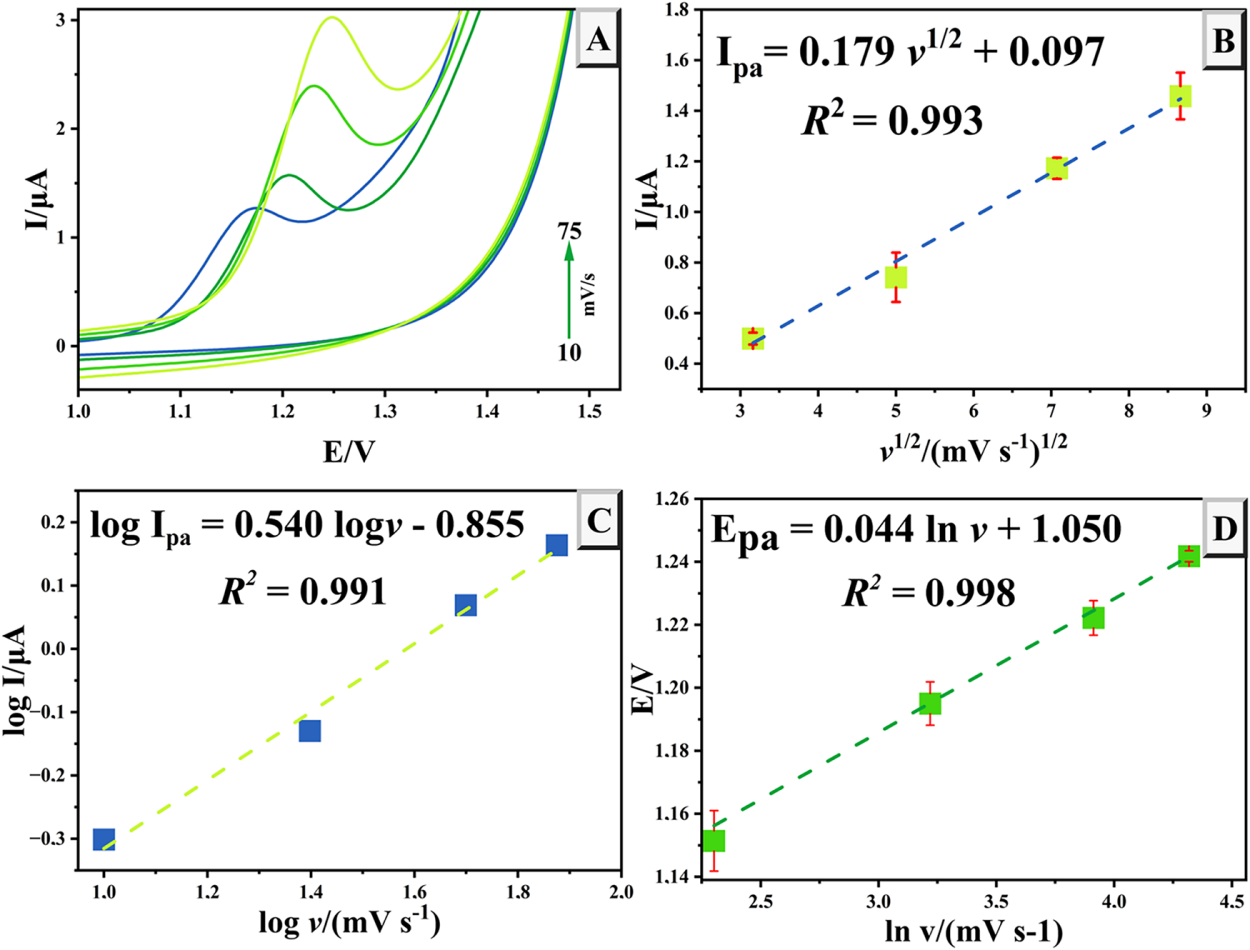
CV curves recorded at scan rates ranging from 10 to 75 mV/s in B–R buffer (pH 2.0) with 50 μM TRP (A); linear relationship between *I*_pa_ and the square root of the scan rate (B); correlation between the log of *I*_pa_ and the log of the scan rate (C); and linear relation between *E*_pa_ and the natural logarithm of the scan rate (D) at the surface of NiPB@Cu/Cu_2_O/GCE.

### 3.5 Calibration Plot and Limit of Detection for TRP

By employing DPV approach under optimized conditions, a calibration curve was established across a broad concentration range of 1.0–17.3 μM, as illustrated in Figure 5A. Measurements of TRP solutions at varying concentrations were performed on the modified GCE surface, yielding a strong linear correlation between the TRP oxidation peak currents and their concentrations within this range. The linear relationship was expressed by the equation *I* = 0.226*C*_TRP_ – 0.147 with a correlation coefficient of 0.997 (Figure 5B). The detection limit (LOD) and quantification limit (LOQ) were determined to be 0.03 μM and 0.1 μM, respectively, demonstrating the high sensitivity and reliability of the proposed sensor.

**Figure 5:**
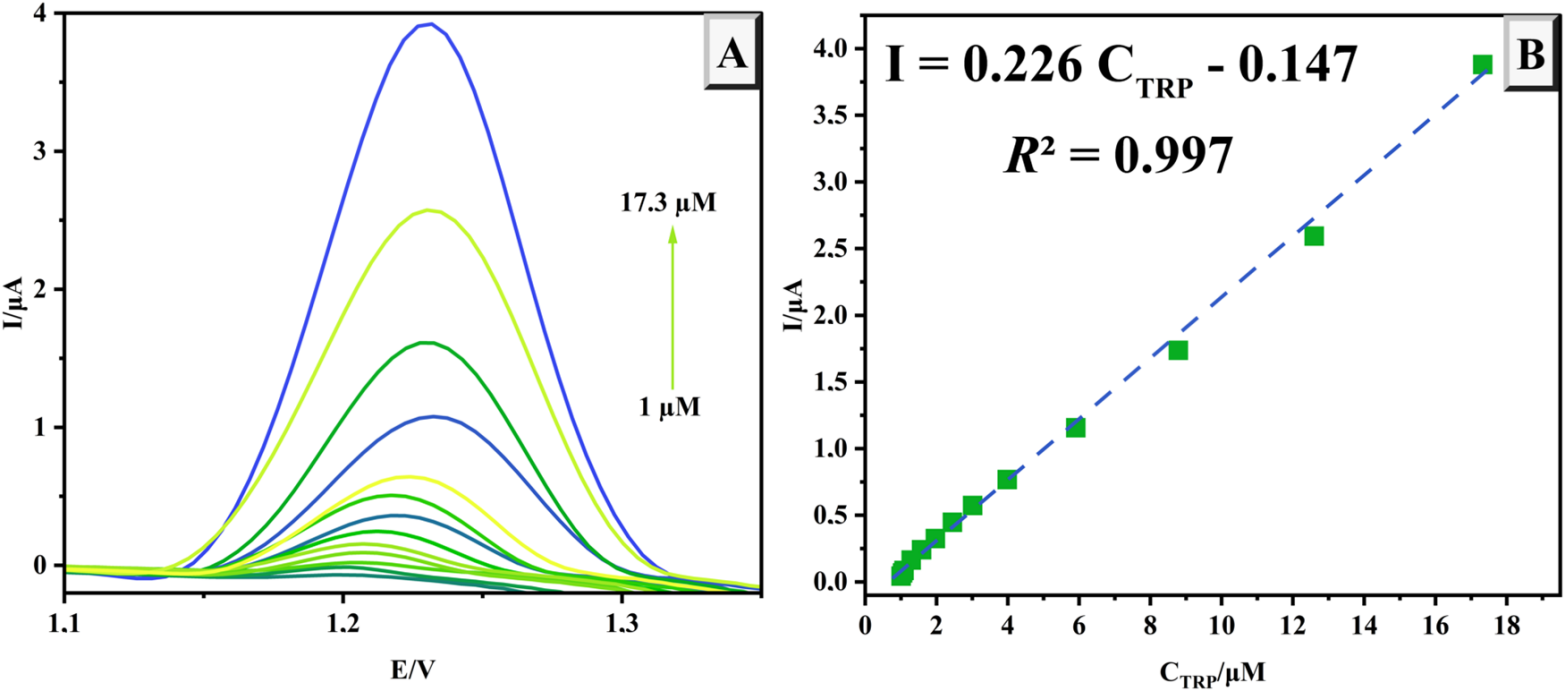
DPV signals of the NiPB@Cu/Cu_2_O/GCE at various TRP concentrations (A) and the plot of *I*_pa_–*C*_TRP_ (B).

As summarized in Table 2, the proposed DPV-based sensor provides competitive analytical performance compared to previously reported methods for TRP quantification. While the LC-MS/MS method offers ultratrace detection suitable for pharmacokinetic studies, it demands costly instrumentation, extensive sample preparation, and expert handling. Similarly, RP-HPLC techniques, although effective in pharmaceutical formulations, are limited by their lack of compatibility with complex biological matrices and generally require long run times and high solvent consumption.

**Table 2:** Comparison of Analytical Methods for TRP Determination

method	linear range	LOD (μg/mL)	LOQ (or LLOQ)	application	recovery (real or spiked samples, %)	refs
LC-MS/MS	0.25–75 ng/mL		0.25 ng/mL	rat serum, human serum, and human plasma	104.25–110.66	^14^
RP-HPLC	5–30 ppm	0.05	0.14 μg/mL	treprostinil sodium solution	99.34–99.70	^16^
RP-HPLC	2.5–15 μg/mL	0.12	0.38 μg/mL	treprostinil solution	99.24–101.89	^15^
DPV	1–17.3 μg/mL	0.03	0.1 μM	human urine, human plasma, and pharmaceutical sample	99.27–102.24	our work

In contrast, the present electrochemical sensor achieves excellent recovery rates in plasma, urine, and pharmaceutical samples with minimal pretreatment. The sensor offers a low-cost, environmentally friendly, and portable alternative that enables rapid detection within minutes. These features make it especially valuable for point-of-care diagnostics and routine quality control in pharmaceutical settings, where accessibility and simplicity are prioritized over extreme sensitivity. Therefore, this study not only introduces the first voltammetric approach for TRP detection but also presents a practical platform capable of bridging the gap between high-end laboratory methods and real-world field applications.

### 3.6 Repeatability and Reproducibility of the Sensing Platform

For the repeatability assessment, nine consecutive differential pulse voltammetry (DPV) measurements were conducted in a 5 μM TRP solution using the NiPB@Cu/Cu_2_O/GCE. The obtained relative standard deviation (RSD) value was 2.96%, as depicted in Figure S7. The minimal variation in peak currents across successive measurements confirms the high repeatability of the developed sensor, indicating its stability during repeated use. Additionally, to evaluate the reproducibility of the sensor, eight independently fabricated electrodes were tested under identical conditions (Figure S8). The resulting RSD value of 2.50% demonstrates the high reproducibility of the sensor, further highlighting its reliability and potential applicability in real-world electrochemical sensing.

### 3.7 Selectivity of NiPB@Cu/Cu_2_O/GCE

The fabricated NiPB@Cu/Cu_2_O/GCE is anticipated to exhibit a selective signal to the target analyte, effectively differentiating it from potential interferents. Thus, selectivity plays a crucial role in determining the accuracy and reliability of the electrochemical sensor. To evaluate the selectivity of the sensor, various common interfering substances were introduced, such as ascorbic acid (AA), uric acid (UA), d-glucose (DG), l-arginine (LA), l-methionine (LM), potassium chloride (KCl), sodium sulfate (Na_2_SO_4_), potassium nitrate (KNO_3_), dopamine (DOPA), caffeine, and paracetamol (Figure S9). These compounds were selected based on their presence in biological and pharmaceutical samples, as well as their potential to interfere with electrochemical measurements.

Ascorbic acid (AA) and uric acid (UA) are naturally occurring redox-active compounds commonly found in biological fluids, which can generate overlapping signals and interfere with electrochemical detection. Dopamine (DOPA), a neurotransmitter with strong electrochemical activity, is another potential interferent, particularly in biological sensing applications. d-Glucose (DG), a major component in blood and other physiological fluids, is included to assess the sensor’s performance in complex biological matrices. Similarly, amino acids such as l-arginine (LA) and l-methionine (LM) are tested due to their ability to adsorb onto electrode surfaces or participate in redox reactions, potentially affecting sensor response.

In addition to biological interferents, electrolyte salts, including potassium chloride (KCl), sodium sulfate (Na_2_SO_4_), and potassium nitrate (KNO_3_), are used to examine the influence of ionic strength on the electrochemical response of the sensor. Furthermore, pharmaceutical compounds such as caffeine and paracetamol are included to ensure that the sensor maintains its selectivity in the presence of common drug molecules.

The RSD value was determined to be 1.11%, showing that the NiPB@Cu/Cu_2_O/GCE sensor exhibits excellent selectivity and remains unaffected by the presence of these interfering substances during the determination of 5 μM TRP. These findings confirm the high specificity and reliability of the developed sensor, reinforcing its potential for practical electrochemical sensing applications.

To further assess the selectivity of the proposed sensor under mixed-analyte conditions, DPV measurements were performed for TRP (1.0–5.9 μM) in the presence of dopamine, paracetamol, and uric acid separately. Well-resolved oxidation peaks corresponding to each analyte were observed, confirming the ability of the sensor to simultaneously distinguish TRP from these common interferents. Calibration studies revealed that the LOD values of TRP were 0.047 μM with PARA (1.0–6.0 μM) (Figure S10), and 0.038 μM with UA (1.0–6.0 μM) (Figure S11), and 0.04 μM with DOP (1.0–8.0 μM) (Figure S12), which are comparable to the LOD obtained for TRP alone (0.03 μM). The close similarity between these values demonstrates that even in the presence of excess electroactive interferents, the TRP response remains reliable and selective, underscoring the robustness and specificity of the developed electrode (Table 3).

**Table 3:** Quantification of TRP in Spiked Biological and Pharmaceutical Samples

sample	added (μM)	found (μM)	RSD (%)	recovery (%)
human urine	1.0	1.02	2.67	102.14
	2.0	2.01	2.47	100.57
	3.0	2.89	1.79	99.27
human plasma	1.0	0.98	2.61	102.08
	2.0	2.03	2.98	101.50
	3.0	2.99	0.47	99.78
pharmaceutical sample	1.0	1.00	2.27	100.86
	2.0	1.99	2.49	99.70
	3.0	3.06	1.23	102.24

### 3.8 Stability

The interday stability of the proposed electrochemical sensor was assessed by repeatedly recording the peak current response of the analyte over an extended period of 11 days, with measurements performed every other day (days 1, 3, 5, 7, 9, and 11). The representative differential pulse voltammograms are illustrated in Figure 6A, while the corresponding bar diagram showing the trend of current changes is provided in Figure 6B. The obtained results are summarized in Table 4. The average peak current throughout this period was calculated as 5.05 × 10^–7^ A, with a standard deviation of 2.67 × 10^–8^ A, resulting in an interday precision of 5.28% RSD. Compared with the baseline measurement on day 1 (5.34 × 10^–7^ A), the peak current gradually decreased to 4.75 × 10^–7^ A on day 11, corresponding to an overall reduction of approximately 11%. Although a moderate decline was observed, the variability remained within acceptable limits. According to ICH Q2­(R1) and its updated draft Q2­(R2), as well as FDA and EMA bioanalytical method validation guidelines, interday (intermediate) precision is considered acceptable when %RSD values are below 15% (20% at the lower limit of quantification).^37,42^ In this context, the observed 5.3% RSD clearly confirms that the developed sensor maintains reliable interday stability, and its reproducibility falls well within internationally recognized validation standards.

**Figure 6:**
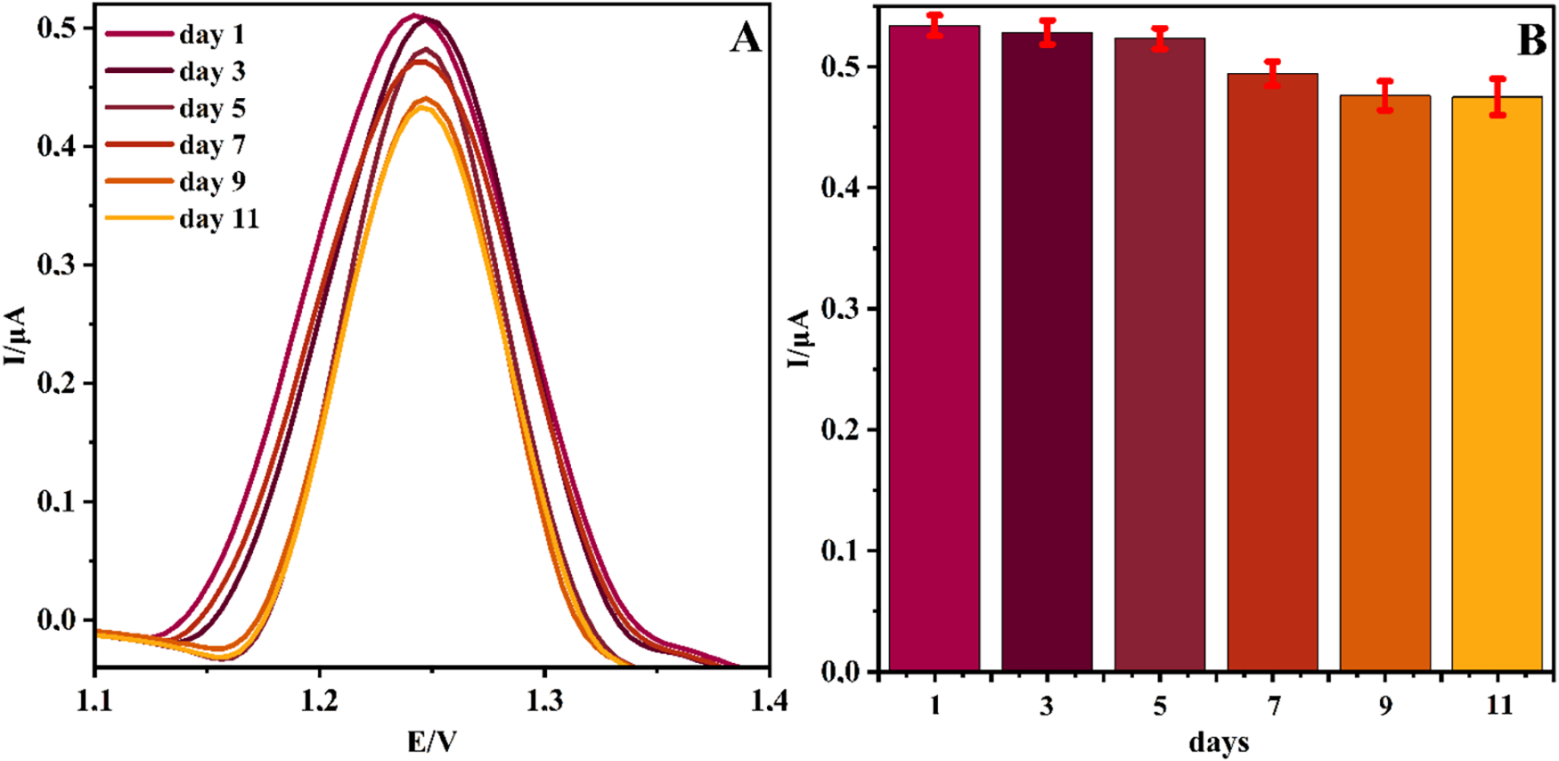
DPV signals of the 50 μM TRP at NiPB@Cu/Cu_2_O/GCE (A) and the corresponding diagrams at different days (B).

**Table 4:** Inter-Day Stability Results of the Fabricated Electrochemical Sensor for CNG Determination over 11 Days (*n* = 6, Measured Every Other Day)

day	peak current (A)	% change vs day 1
1	5.34 × 10^–7^	-
3	5.28 × 10^–7^	1.1%
5	5.23 × 10^–7^	2.1%
7	4.94 × 10^–7^	7.5%
9	4.76 × 10^–7^	10.9%
11	4.75 × 10^–7^	11.0%

### 3.9 Spiked Sample Analysis

To evaluate the performance of the developed electrochemical sensor, TRP was determined in spiked human urine, plasma, and pharmaceutical samples using the standard addition method. The NiPB@Cu/Cu_2_O/GCE sensor, combined with the DPV technique, was employed to ensure precise and reliable TRP determination in these complex matrices. The analytical performance of the sensor was evaluated by calculating recovery rates, which were found to be highly satisfactory. As summarized in Table 3, the recovery percentages varied from 99.27% to 102.14% for urine samples, 99.78% to 102.08% for plasma samples, and 99.70% to 102.24% for pharmaceutical formulations. Furthermore, the method exhibited excellent precision, as reflected in the low relative standard deviation (RSD) values, all of which remained below 2.98%. These findings strongly support the robustness, accuracy, and reproducibility of the proposed voltammetric approach, confirming its suitability for the reliable quantification of TRP in biological and pharmaceutical samples. The high recovery rates, coupled with minimal variations, indicate that the sensor is minimally affected by matrix effects, further demonstrating its potential as a cost-effective and efficient analytical tool for routine TRP monitoring in clinical and pharmaceutical applications.

## 4 Conclusion

This study reports the development of a novel electrochemical sensor for the determination of treprostinil (TRP), based on a glassy carbon electrode modified with a NiPB@Cu/Cu_2_O composite. The sensor exhibited improved current response and lower oxidation potentials compared to the bare GCE, indicating enhanced electron transfer at the modified surface. The electrochemical behavior of TRP was systematically studied using cyclic voltammetry (CV) and differential pulse voltammetry (DPV), with DPV offering higher sensitivity for TRP quantification.

The sensor demonstrated a linear response in the range of 1.0–17.3 μM with a detection limit of 0.03 μM. It also showed acceptable reproducibility and repeatability, as indicated by low relative standard deviation (RSD) values. Selectivity studies showed that the sensor maintained a stable response in the presence of some common coexisting substances, although further work is needed to assess its performance against clinically relevant metabolites and degradation products.

The method was successfully applied to the determination of TRP in spiked human plasma, urine, and pharmaceutical samples using the standard addition method, with recovery rates exceeding 99%. However, it should be noted that the tested concentrations were higher than those typically encountered in clinical practice, and additional optimization is needed for trace-level detection in real matrices. While the current sensor does not yet reach the ultratrace detection limits required for clinical monitoring of TRP, it serves as an initial framework upon which future sensitivity improvements can be built for eventual therapeutic drug monitoring applications.

Although the electrochemical techniques employed in this study (CV and DPV) are widely used in sensor development, their integration with the newly designed NiPB@Cu/Cu_2_O composite has enabled, for the first time, the voltammetric detection of TRP. This application-oriented advancement provides a foundation for future development of rapid, low-cost, and scalable electrochemical methods for prostacyclin analogs, offering an accessible alternative to chromatographic methods for routine analysis.

In conclusion, the NiPB@Cu/Cu_2_O/GCE sensor represents a promising and low-cost approach for TRP determination in controlled laboratory settings. Future studies should focus on improving its applicability at clinically relevant concentrations, validating its long-term stability, and expanding its use to broader electrochemical sensing applications.

## Supplementary Material



## Notes

Additional experimental details include materials, reagents, apparatus, relevant equations, details on the effect of concentration and amount of composite, cyclic voltammograms, and differential pulse voltammograms (PDF)
