# Supplementary material for: Enhanced Electrochemical Sensing of Treprostinil: A Novel Approach for Sensitive and Selective Detection
Source: ACS Omega. 2025 Oct 30;10(44):52507–18. doi: 10.1021/acsomega.5c05604 (PMC12612867; doi:10.1021/acsomega.5c05604)
Supplement: Supplementary file 1 [file ao5c05604_si_001.pdf]

Supporting Information

Enhanced Electrochemical Sensing of Treprostinil: A Novel Approach for Sensitive and Selective Detection

Çiğdem Aybüke Özata[a,b*], ***Nevin Erk***[a*], ***Wiem Bouali***[a,b], ***Asena Ayse Genc***[a,b], ***Furkan Uzcan***[c], ***Mustafa Soylak***[c,d,e]

[a] Ankara University, Faculty of Pharmacy, Department of Analytical Chemistry, 06560 Ankara, Turkey

[b] Ankara University, The Graduate School of the Health Sciences, 06110 Ankara, Turkey

[c] Erciyes University, Faculty of Sciences, Department of Chemistry, 38039, Kayseri, Turkey

[d] Technology Research & Application Center (TAUM), Erciyes University, 38039, Kayseri, Turkey

[e] Turkish Academy of Sciences (TUBA), Cankaya, Ankara, Turkey

Emails of the corresponding authors: erk@pharmacy.ankara.edu.tr

caozata@ankara.edu.tr

Materials and Reagents

In this study, Glucose (99.5 %), L-arginine (98.0 %), L-methionine, sodium hydroxide, potassium hexacyanoferrate (III) ($K_3Fe(CN)_6$, 99.5 %), hydrochloric acid, sodium acetate, ascorbic acid, uric acid (99.0 %), potassium chloride, sodium phosphate, sodium sulfate, potassium chloride, sodium sulfate, and ethanol were purchased from Sigma Aldrich Co. (https://www.sigmaaldrich.com, Germany). Britton-Robinson buffer was made of boric acid, phosphoric acid, potassium chloride, and acetic acid solutions. The stock solution of Treprostinil was prepared in pure distilled water. All chemical compounds were analytical grade and used without additional refinement.

Apparatus

Voltammetric experiments were carried out using AUTO LAB system with PGSTAT204 electrochemical workstation (Metrohm Inc., Switzerland) with a glassy carbon electrode system in a one-compartment of 10 mL electrochemical cell. All electrochemical measurements were performed at 25 °C unless otherwise specified.

The Randles-Sevcik equation:

$$I_p = (2.69 \times 10^5)\, n^{\frac{3}{2}}\, A\, D^{\frac{1}{2}}\ v^{\frac{1}{2}}\, C_0 \qquad (S1)$$

Symbol	Parameter Description	Unit
I_p	Peak current	A (amperes)
n	Number of electrons transferred	–
A	Electroactive surface area	cm^2
D	Diffusion coefficient (for $[Fe(CN)_6]^{3-}/^{4-}$ typically 7.6×10^{-6})	cm^2/s
C_0	Concentration of the redox species	mol/cm^3
v	Scan rate	V/s

- The peak current values (Ip) were obtained from cyclic voltammograms recorded at different scan rates.
- The slope was obtained from the linear plot of Ip versus $v^{1/2}$, as shown in Figure 4B.
- The electroactive surface area (A) was calculated by rearranging the Randles–Ševčík equation using the experimentally derived slope.

$$R_{ct} = \frac{RT}{F^2\,C\,A\,k^0} \qquad (S2)$$

$$R_{ct} = \frac{RT}{n\,F\,A\,j_0} \qquad (S3)$$

k^0 represents the standard heterogeneous electron transfer rate constant (cm s^{-1}), j_0 is the exchange current density (A cm^{-2}), R stands for the universal gas constant (8.314 J K^{-1} mol^{-1}), T is the temperature (298.15 K), F is the Faraday constant (96485 C mol^{-1}), R_{ct} is the electron transfer resistance (Ω), A is the electrode surface area (cm^2), n is the number of electrons transferred, and C is the concentration of the $[Fe(CN)^6]^{3-/4-}$ solution (5×10^{-6} mol cm^{-3}).

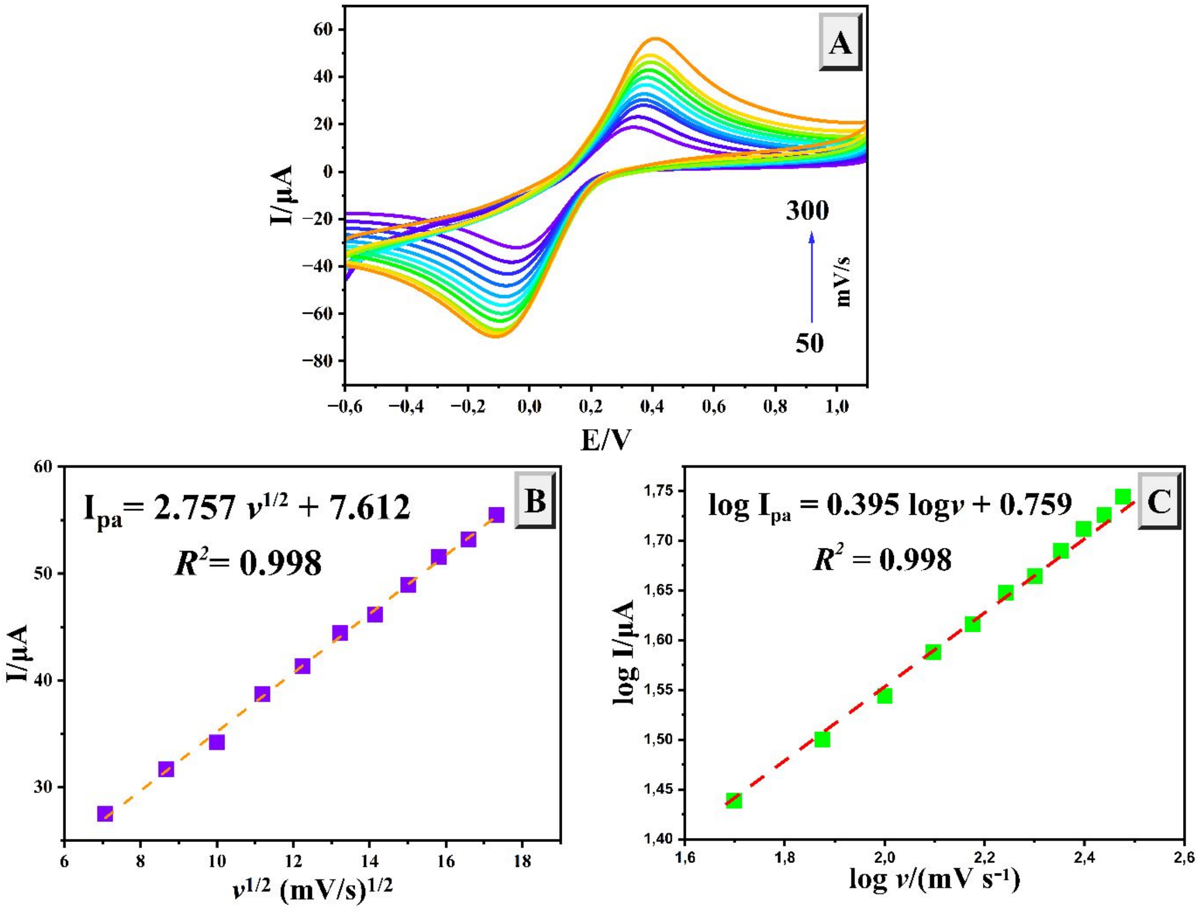

Figure S1. The recorded CV curves (A), the relationship between I_{pa} vs. $v^{1/2}$ (B), and the relationship between logI_{pa}-log$v$ (C) on the bare GCE at various scan rates in 5.0 mM $[Fe (CN)_6]^{3-/4-}$ and 0.1 M KCl.

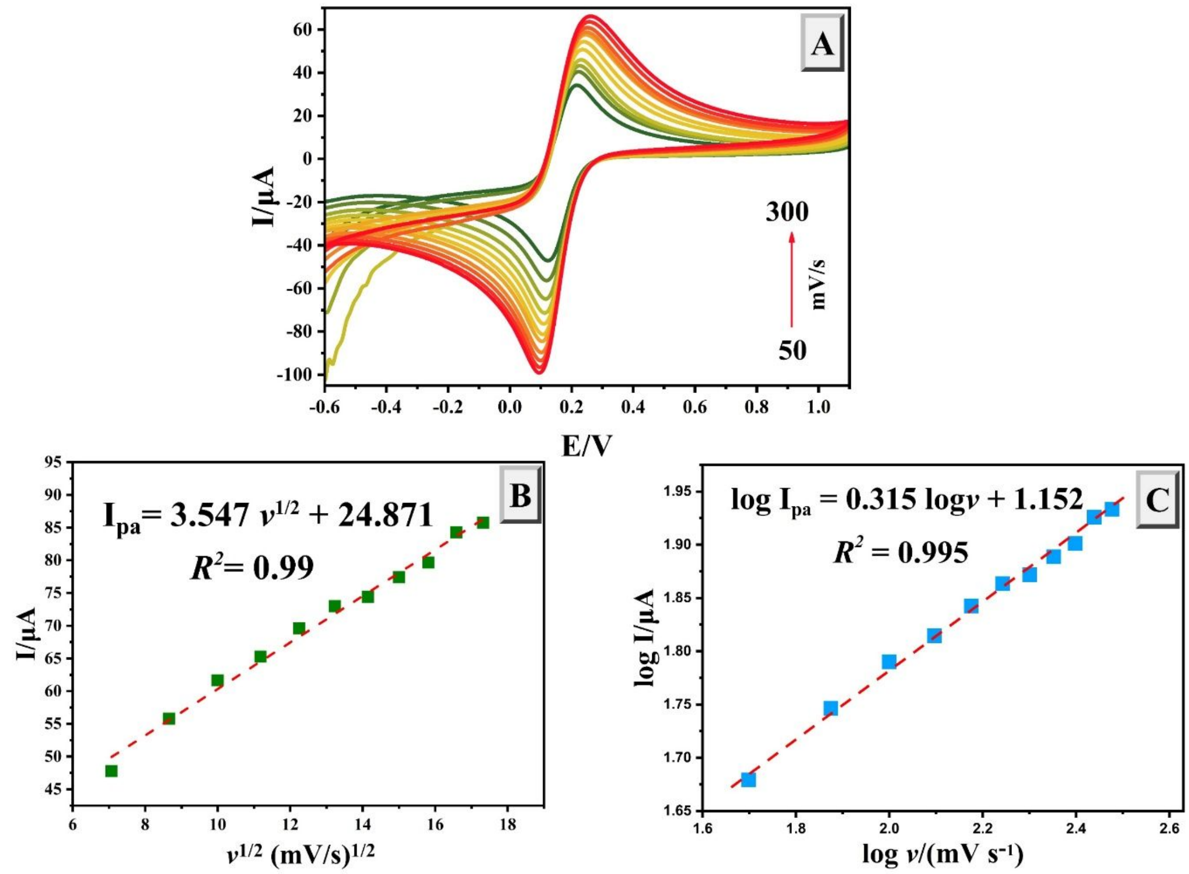

Figure S2. The recorded CV curves (A), the relationship between I_{pa} vs. $v^{1/2}$ (B), and the relationship between logI_{pa}-log$v$ (C) on the NiPB@Cu/Cu_2O/GCE at various scan rates in 5.0 mM $[Fe(CN)_6]^{3-/4-}$ and 0.1 M KCl.

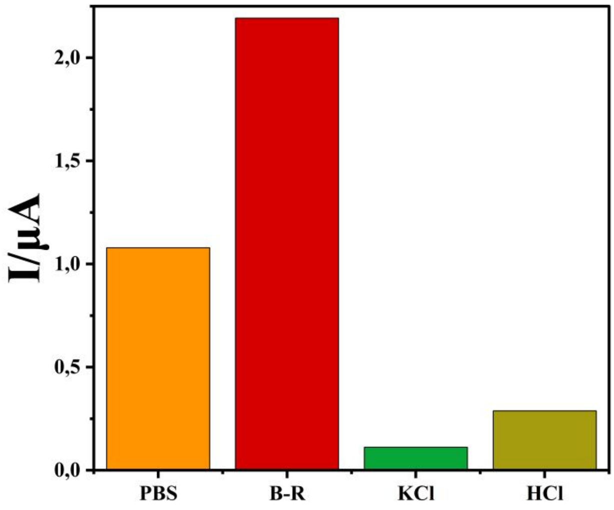

Figure S3. Influence of supporting electrolyte.

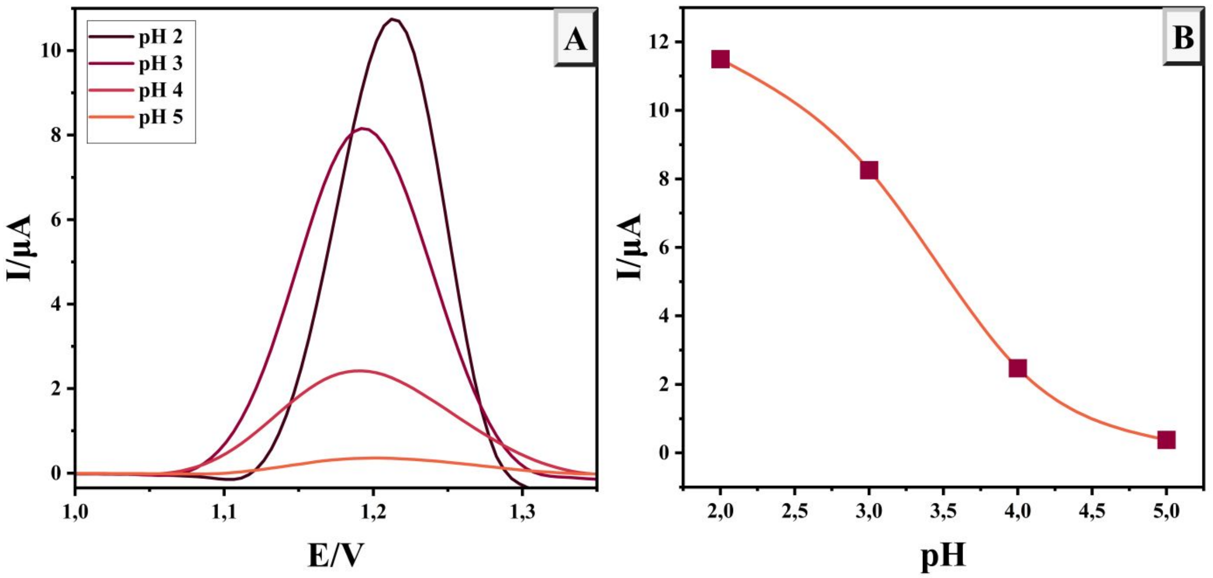

Figure S4. DPVs of 50 µM TRP on NiPB@Cu/Cu_2O/GCE in B-R buffer at different pH values (A), and the plot of Ip of TRP versus pH (B).

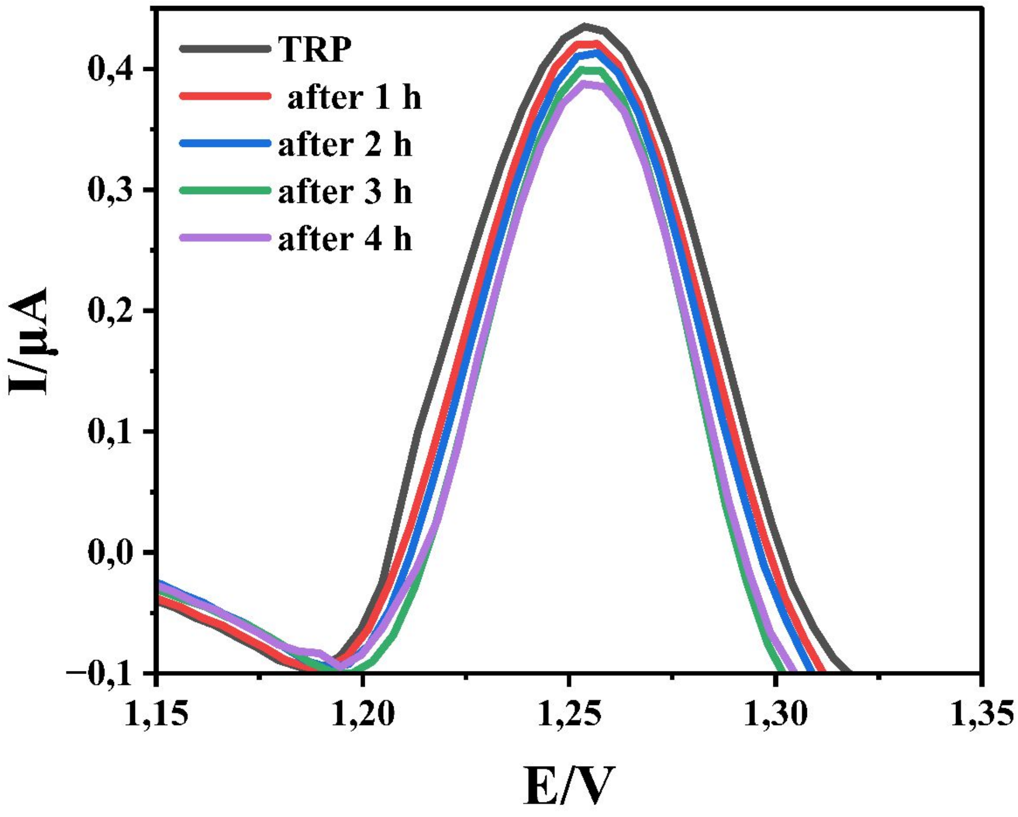

Figure S5. Stability of TRP in B-R buffer at pH 2 for 4 hours.

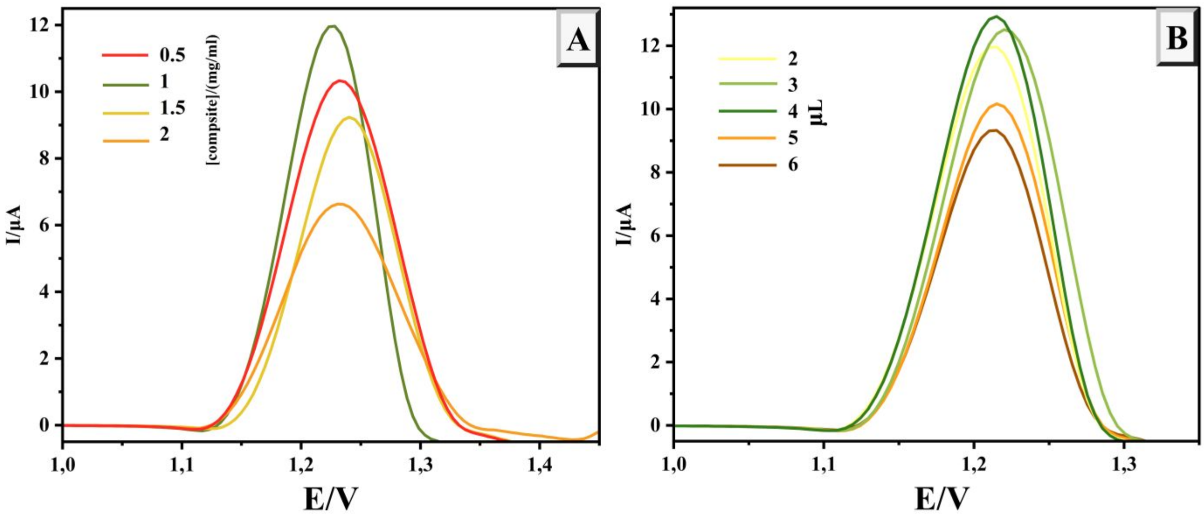

Figure S6. Composite concentration (A) and quantity (B) on the oxidation peak currents of 50 μM TRP at the surface of NiPB@Cu/Cu_2O/GCE.

Laviron's Theory :

$$Epa = \frac{RxT}{\alpha xFxslope} \qquad (S4)$$

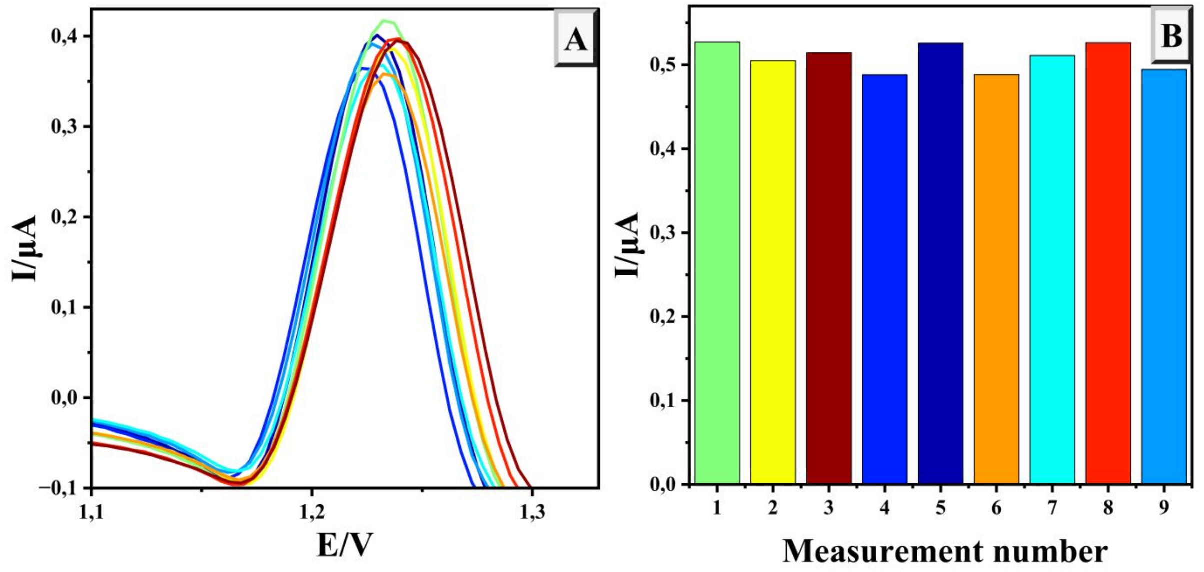

Figure S7. DPV curves and histogram of repeatability of NiPB@Cu/Cu_2O/GCE in 5 μM TRP (B-R buffer, pH 2.0).

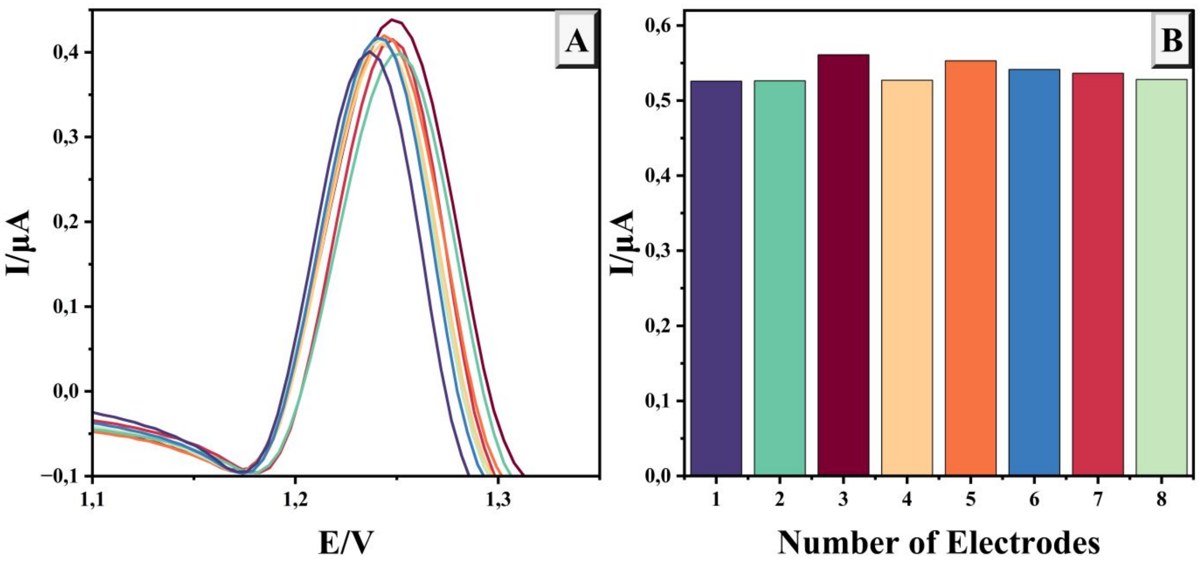

Figure S8. DPV curves and histogram of reproducibility of NiPB@Cu/Cu_2O/GCE in 5 μM TRP (B-R buffer, pH 2.0).

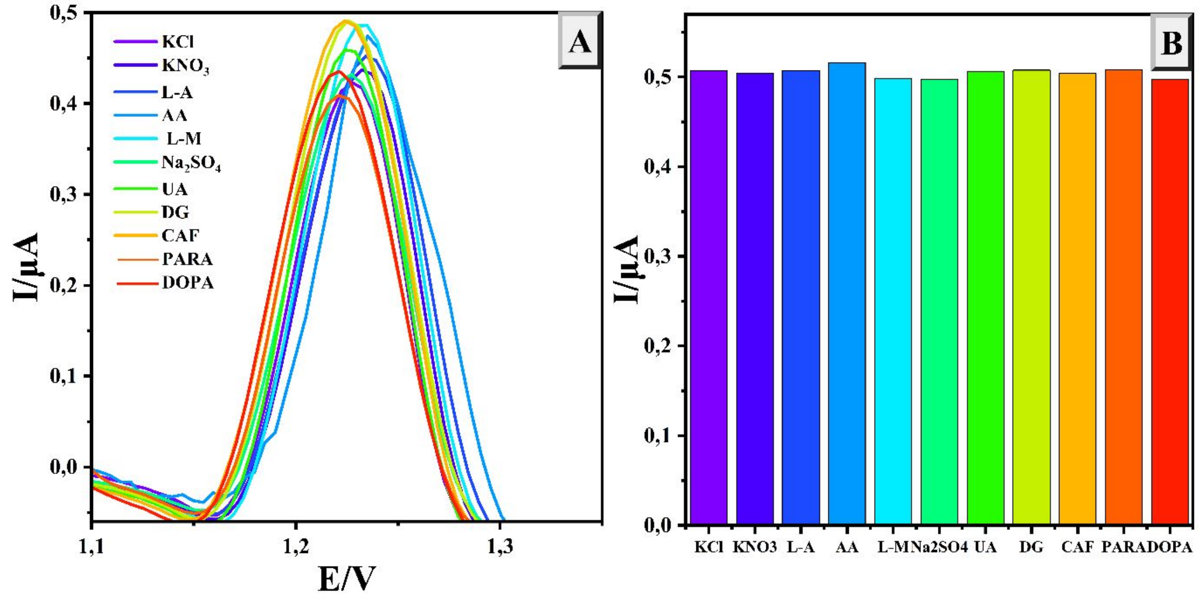

Figure S9. DPV curves and histogram of selectivity of 5 μM TRP at NiPB@Cu/Cu_2O/GCE in B-R buffer, pH 2.0.

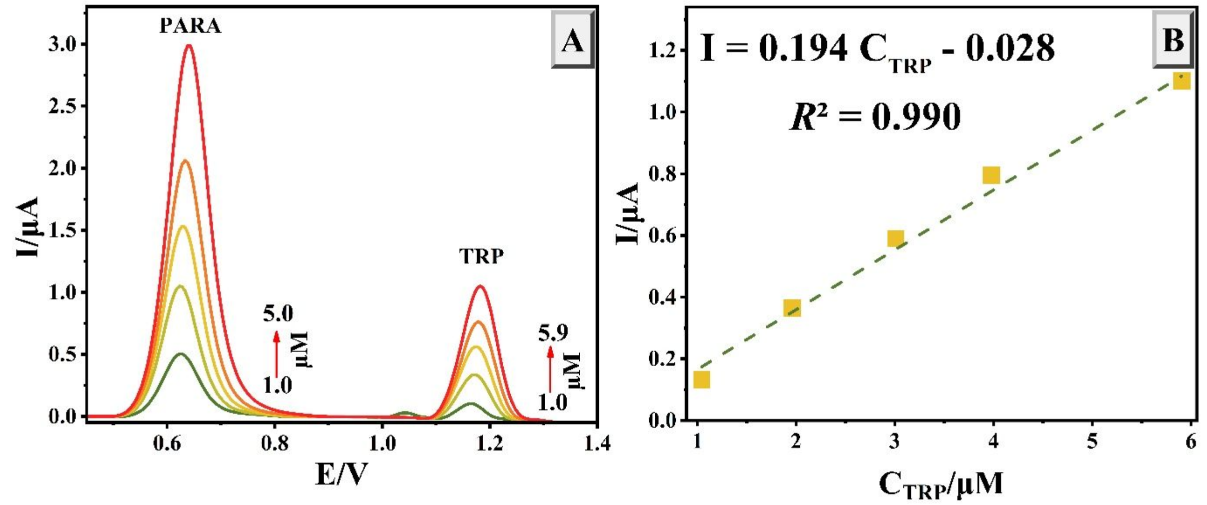

Figure S10. (A) DPVs of different concentrations of PARA (1.0-5.0 µM) and TRP (1.0-5.9 µM), and (B) plot of Ipa (µA) vs. conc. of TRP at NiPB@Cu/Cu_2O/GCE in B-R buffer, pH 2.0.

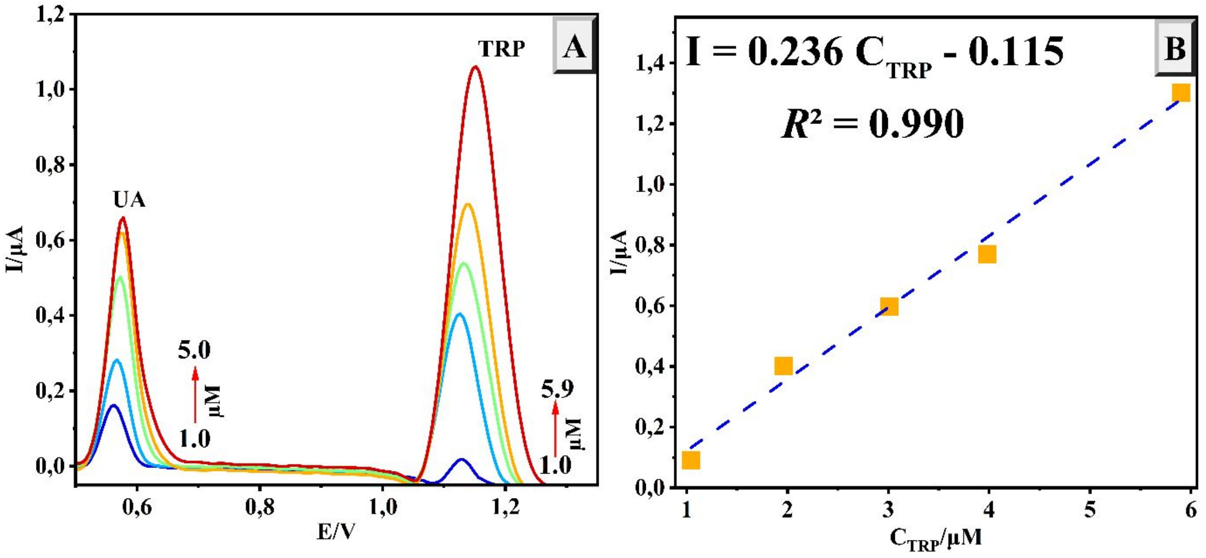

Figure S11. (A) DPVs of different concentrations of UA (1.0-5.0 µM) and TRP (1.0-5.9 µM), and (B) plot of Ipa (µA) vs. conc. of TRP at NiPB@Cu/Cu_2O/GCE in B-R buffer, pH 2.0.

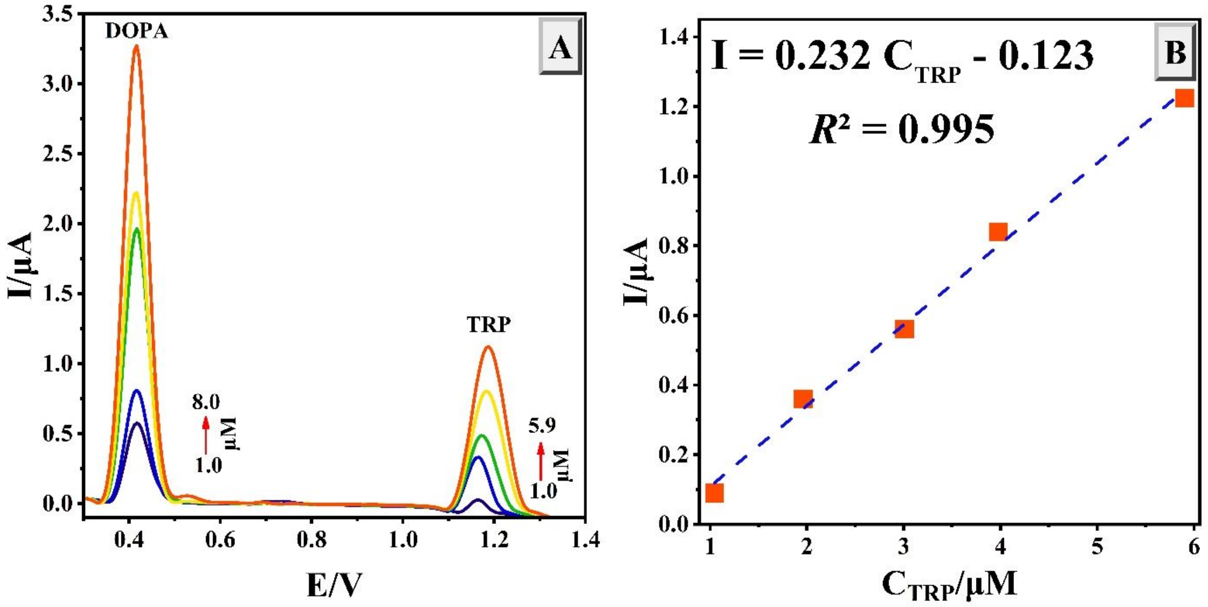

Figure S12. (A) DPVs of different concentrations of DOP (1.0-8.0 μM) and TRP (1.0-5.9 μM), and (B) plot of Ipa (μA) vs. conc. of TRP at NiPB@Cu/Cu_2O/GCE in B-R buffer, pH 2.0.
